# Transactions of the Medical Association of Georgia

**Published:** 1883-06

**Authors:** 


### TRANSACTIONS OF THE MEDICAL ASSOCIATION OF GEORGIA.

It would have been greatly to the interest of our readers, and accordingly gratifying to us, had it been possible to make a heavier transfer to the pages of the REGISTER from those of the Transactions.

We could only publish in full the admirable address of President Holt, of Macon, and the section report of Dr. T. S. Dekle. There are several other papers of conspicuous merit, which we shall reserve for a future notice:

The Annual Oration by J. P. Stevens, M.D., of Macon.

Report of Surgery for the Second Congressional District, by P. L. Hilsman, M.D., of Albany.

Fistula in Ano—With Report of Three Cases, by L. M. Jones, M.D., of Gordon.

Relative Merits of Humanized Bovine Vaccine Virus, by Eugene Foster, M.D., President of Board of Health, Augusta.

Sympathetic Inflammation—Its Mode of Transmission and Its Nature, by J. M. Hull, M.D., of Augusta.

Syphilitic Ulceration of the Eye-Lid (Conjunctiva) in the Infant, by A. W. Calhoun, M.D., of Atlanta.

The Use of Ergot in Chronic Affections of the Ear,

Throat and Lungs—With Experiments and Report of Cases, by H. F. Scott, M.D., of Atlanta.

Is Typhoid Fever Contagious? by W. H. Philpot, M.D., of Talbotton.

Notes from the Fourth Congressional District, by T. S. Mitchell, M.D., of Hamilton.

Report of a Case of Complete Atresia of Cervix Uteri, With Retention of Menses—Produced by Nitric Acid—Relieved by Aspiration and Bilateral Division of the Cervix, by E. H. Richardson, M.D., of Cedartown.

Report on Gynecology, by C. P. Gordon, M.D., of Dalton.

Malarial Poisoning—The Cause of Hæmaturia, by W. O'Daniel, M.D., of Bullards.

